# Predictive Model of Nail Consistency Using Scanning Electron Microscopy with Energy-Dispersive X-Ray

**DOI:** 10.3390/biology10010053

**Published:** 2021-01-12

**Authors:** Esther Mingorance Álvarez, Rodrigo Martínez Quintana, Ana Mª Pérez Pico, Raquel Mayordomo

**Affiliations:** 1 Department of Anatomy and Cell Biology, University Center of Plasencia, University of Extremadura, Avda. Virgen del Puerto 2, 10600 Plasencia, Cáceres, Spain; emingorance@unex.es; 2 Department of Mathematics, University Center of Plasencia, University of Extremadura, Avda. Virgen del Puerto 2, 10600 Plasencia, Cáceres, Spain; rmartinez@unex.es; 3 Department of Nursing, University Center of Plasencia, University of Extremadura, Avda. Virgen del Puerto 2, 10600 Plasencia, Cáceres, Spain; aperpic@unex.es

**Keywords:** binary logistic regression, nail apparatus, nail consistency, nail plate, predictive model, SEM-EDS, thickness

## Abstract

**Simple Summary:**

Scanning electron microscopy with energy-dispersive X-ray spectroscopy (SEM-EDS) is a useful technique to analyse elemental composition in the nail plate. The dorsal, intermediate, and ventral layers are differentiated by the levels of the elements present in each layer. The level of calcium in the dorsal layer is the main predictive variable in calculating the predictive model of consistency. This model will provide further knowledge of the factors that determine nail consistency in individuals and help health professionals to better understand nail characteristics and objectively determine nail consistency.

**Abstract:**

The nail plate is made up of tightly packed keratin-rich cells. Factors such as the special distribution of the intermediate filaments in each layer (dorsal, intermediate, and ventral), the relative thickness of the layers, and their chemical composition define the characteristics of each nail. The main objective of this study is to determine nail consistency by calculating a predictive model based on elemental composition analysis using scanning electron microscopy. Nail consistency was determined in 57 participants (29 women and 28 men) in two age groups (young people and adults). Elemental composition was analysed in each layer using scanning SEM-EDS, and nail plate thickness was measured by image analysis. A total of 12 elements were detected in nail plates, of which carbon, nitrogen, phosphorus, sulphur, and calcium showed significant differences between layers (*p*-values ≤ 0.01). The level of calcium in the dorsal layer was the main predictive variable in calculating the predictive model of consistency, with 75.4% correctly classified cases. Elemental analysis in each layer of the nail plate by SEM-EDS can be used to develop a predictive model of nail consistency that will help health professionals to objectively determine nail consistency.

## 1. Introduction

The human nail apparatus is a structure of epidermal origin located on the dorsal plane of the distal phalanges of toes and fingers. The anatomy of the human nail apparatus has been widely studied, and in addition to the nail plate, it comprises periungual soft tissues, with vascularisation and innervation [1,2,3].

**Figure 1:**
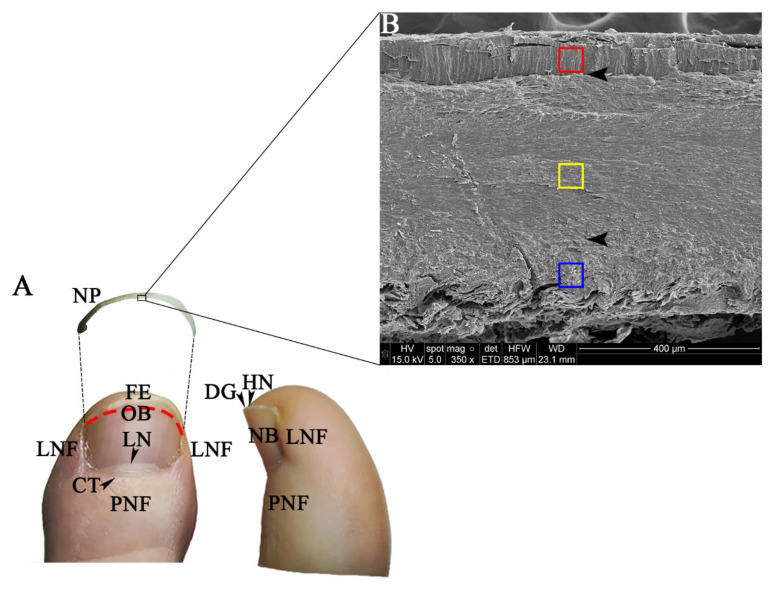
Nail apparatus and measuring areas in the nail plate. (**A**) Dorsal and medial view of the first toe of the left foot and elements of the nail apparatus identifiable to the naked eye. CT, cuticle; DG, distal groove; FE, free edge; HN, hyponychium; LN, lunula; LNF, lateral nail fold; NB, nail bed; NP, nail plate; OB, onychodermal band (red discontinuous line); PNF, proximal nail fold. (**B**) Scanning electron micrograph in high vacuum conditions to illustrate the structure of human nail plate and the measuring areas selected. The dorsal area (red), intermediate area (yellow), and ventral area (blue) layers are shown. Arrowheads indicate the limit between the nail plate layers.

Both the proximal nail fold and the two lateral nail folds are cutaneous folds situated in the most proximal region and on both sides of the nail plate, respectively. The proximal fold is located on the nail matrix and continues to the cuticle (eponychium), the translucent stratum corneum that secures the proximal fold to the nail plate, while the lateral nail folds border both sides, providing support to the structure and guiding the correct growth of the nail plate. The nail matrix is the epithelial germinative structure of the nail plate and extends to the lunula. The nail bed and the nail matrix are characterised by the absence of a granular layer. The lunula is the part of the distal nail matrix visible through the nail plate. It is crescent-shaped and normally whiter than the adjacent nail bed tissue. The nail bed is the pink area under the nail plate, to which it is strongly attached. It goes from the distal end of the nail matrix or lunula to the hyponychium and contains the onychodermal band. This band is usually a more pink or reddish colour and forms a barrier that prevents material from entering under the nail plate. The hyponychium extends from the distal end of the onychodermal band to the distal groove that separates the nail bed from the tissue of the finger pulp. The nail plate is the slightly curved structure that covers the dorsal surface of the distal digit, which it protects [1,2,3,4,5] (Figure 1).

The nail plate is made up of tightly packed, anucleate keratinised cells [1,2,3,4,5] and is a structure that grows continually throughout an individual’s lifetime [5]. Keratin is included in the group of scleroproteins and is the most abundant intermediate filament in the nail plate, with a very stable folded α-helical structure formed by disulphide bonds (cystines) [5,6]. The human genome codifies for 54 functional keratin genes and 13 pseudogenes, grouped into two clusters on chromosomes 17q21.2 and 12q13.13 [7]. Keratins are classified by their location as epithelial (typical of epithelial tissue) and hair keratins (present in hair and nails). Human keratins are grouped by their genetic sequence into type I acids (epithelial: K9–10, K12–K20, K23–28; hair: K31–K40) and type II basic or neutral (epithelial: K1–K8, K71–K80; hair: K81–K86) [7] and always form heterodimers [8]. Immunohistochemical analysis has made it possible to localise the expression of hair and epithelial keratins in the different components of the nail apparatus [9,10]. Surprisingly, 10%–20% of the nail plate is epithelial keratin [10,11]. The amino acids of the hair keratins present in healthy nail plates [12] vary depending on whether they are located in the head, rod, or tail domains, although cysteine and glutamic acid make up a high percentage of the total amino acid sequence. Glutamate, leucine, alanine, and glutamine are much more abundant in the rod domain, and serine and glycine are found more frequently in the head and tail domains [13]. In accordance with the amino acid content of keratins, the concentrations of the most abundant (carbon, oxygen, nitrogen, and sulphur) and least abundant (calcium, magnesium, aluminium, silicon, and sodium) elements present in the nail plate were determined through scanning electron microscopy (SEM) with energy-dispersive X-ray spectroscopy (EDS) [14]. This analytical technique is very versatile and can be used to determine the elemental composition of a surface provided the atomic number is >5 [15].

The nail plate starts in the nail matrix and grows out continuously over the nail bed, unlike other cutaneous appendages such as hair, which grows in cycles and alternates between periods of growth and rest [5]. The mean thickness of healthy fingernails is approximately 0.5 mm [5,16], while healthy toenails are known to be thicker (1–1.8 mm) [17,18]. Nail thickness, at the onychodermal band, is determined by several factors, including age, the initial thickness of the proximal nail matrix, the length of the lunula (nail matrix), and the length of the nail bed [19].

Histologically, three layers are distinguished in the nail plate: the dorsal, intermediate, and ventral layers [5,20,21]. The intermediate layer is the thickest [5,20,21], with a thickness ratio of 3:5:2 [21]. The orientation of the keratin filaments in the cells of each layer varies. In the dorsal and ventral layers, the orientation is random, but in the intermediate layer it is transverse, parallel to the lunula. The orientation determines the relative toughness of each layer and the fracture properties of nails in the transverse and longitudinal direction [20].

Consistency applied to the nail plate is a recent, little studied concept. It was used to clinically define one of the most prevalent nail disorders, brittle nails, as alterations of nail consistency, characterised by “weakness, inelasticity, flaking, splitting, crumbling, and overall fragility of the nail plate” [22]. The term “nail consistency” arose from routine podiatry practice and the need to determine whether ingrown toenails, another prevalent nail disorder, was associated with consistency [23]. A method was established to qualitatively determine nail consistency in vivo by applying pressure manually to the nail edges. This made it possible to standardise the examination procedure for nail consistency. Using this method, three types of nail consistency were identified in different samples: soft-consistency nails, medium-consistency nails, and hard-consistency nails [23,24]. Hard-consistency nails are more frequent in adult males, whereas in adult women medium-consistency nails predominate [24]. In young individuals medium-consistency nails predominate, although in young runners, hard-consistency nails represent the most frequent type [23].

In view of this, many variables can be included in the definition of nail consistency: the anatomy of the nail apparatus in general and of the nail plate in particular; the specific molecular composition of each nail plate, defined by all the keratins expressed in each individual; the elemental composition; the thickness of the nail plate and its histology and microstructure, etc. As a first step in the study of nail consistency, the main objective of this study was to determine nail consistency through a predictive model developed using elemental composition data obtained in healthy toenails. This will provide further knowledge of the factors that determine nail consistency in every individual.

## 2. Materials and Methods

### 2.1. Permission and Sample Description

All participants signed an informed consent form, and permission for the study was obtained from the University of Extremadura Bioethics Committee (Reg. 116/2016). It was also performed in accordance with the ethical standards laid down in the 1975 Declaration of Helsinki and all subsequent revisions. A physical examination was conducted of participants’ feet and a questionnaire was filled in to obtain the necessary health information. The inclusion criteria were as follows: participants had to be of legal age, not have a diagnosed disease that could alter nail structure and/or composition, not be taking medication described as altering nail structure and/or composition, and be following a Mediterranean diet.

To develop the model, the sample comprised 57 individuals (29 women and 28 men) divided into two age groups: 30 young people (21.50 ± 2.87 years) and 27 adults (50.11 ± 3.43 years) (Table 1). Two individuals were chosen to test the model: an adult male non-smoker with soft-consistency nails and an adult female smoker with hard-consistency nails.

### 2.2. Nail Consistency and Sample Collection

Nail consistency was determined in vivo by trained personnel, applying pressure manually to the nail edges in the lateral–medial and dorsal–ventral axes after exposing the foot to room temperature for 15 min, following the methodology described elsewhere [23].

Samples were collected using the same nail clipper, always on the free edge of the first toe, without exceeding the onychocorneal band. Nail clippings were cleaned by immersion in an ultrasonic cleaning bath (Elma, Singen, Germany) to remove skin cells, fibre, and other debris under the nail plate. Samples were stored at −20 °C until analysis.

### 2.3. SEM-EDS and Thickness Determination

For the elemental analyses, a semi-quantitative analytical technique by capture of dispersed electrons was applied. The microscope used (Quanta 3D FEG, FEI, Eindhoven, The Netherlands) was equipped with one detector (BSED, FEI, Eindhoven, The Netherlands) to obtain images and another detector (Octane Elect Plus, AMETEK, Mahwah, NJ, USA) for the elemental analysis. Samples were attached to aluminium stubs with double-sided carbon tape. For biological samples to withstand high vacuum conditions, they must be subjected to pre-treatments such as chemical fixation, dehydration, freeze-drying or critical point drying, and metal coating with gold or carbon to reduce their water content and allow electricity to be conducted through their tissue. However, these procedures can modify the results of the elemental analysis (coating) or produce artifacts (chemical fixation, dehydration, freeze-drying) [25]. Due to the low water content of nail plates and the equipment used, it was possible to analyse the samples in low vacuum conditions without having to subject them to these types of treatments. The micrographs allowed overall morphological analysis of each sample and identification of the area in each layer of the nail plate for subsequent elemental analysis. They provided no information about the elemental analysis. The image capture conditions were as follows: high voltage (HV) = 20 kV, spot = 6.50–7.50, working distance (WD) = 8.70–11.80 mm, detector (det) = BSED, magnification (mag): 250×, dwell time = 5 µs, aperture diameter = 40 µm, and beam current = 1.71–6.83 nA. The elemental analysis was performed in conditions of low vacuum (100–120 Pa) in an atmosphere of water vapour: accelerating voltage = 20 kV, inclination (tilt) = 0, take-off angle = 33.80–36.40°, detector (det) = octane elect plus, resolution (res) = 125 eV, amplification time (amp. T) = 3.84 µs, FS = 2351–65,222 fs, live time second (Lsec) = 26–185 s, dwell time = 500 ns. The standard ZAF correction method (Z: atomic number, A: absorption, F: fluorescence) was applied. This technique enables determination of the WT% (weight percentage: the weight of the element measured in the sample divided by the weight of all elements in the sample multiplied by 100) and AT% (atomic percentage: the number of atoms of the element, at that weight percentage, divided by the total number of atoms in the sample multiplied by 100) of all elements with an atomic number >5. Thus, the only elements not detected using this technique are hydrogen, helium, lithium, and beryllium.

**Figure 2:**
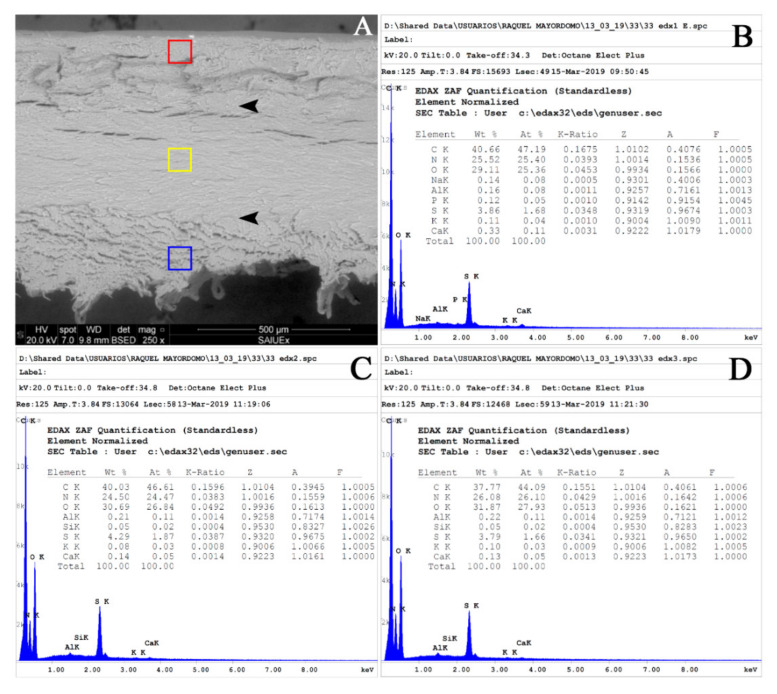
Measuring areas and SEM-EDS in sample 33. (**A**) Scanning electron micrograph in low vacuum conditions to determine the areas for performing the elemental analysis. (**B**) EDS spectrum and elemental quantification table for the dorsal layer. (**C**) EDS spectrum and elemental quantification table for the intermediate layer (**D**). EDS spectrum and elemental quantification table for the ventral layer.

Measurements were made on the surface opposite the free edge of each sample, i.e., on the surface obtained with the clipper, performing a triple analysis (dorsal, intermediate, and ventral layers) (Figure 1). The measuring areas were delimited on scanning electron micrographs in low vacuum conditions (Figure 2), taking into account both the visual limits discernible in the micrographs and the published data of a thickness ratio of 3:5:2 [21].

Nail plate thickness was measured by image analysis, using ImageJ software (http://rsbweb.nih.gov/ij/) on SEM micrographs. Ten random measurements were made on each sample, from the dorsal surface to the ventral surface, and the mean and standard deviation were calculated.

### 2.4. Statistics

Statistical treatment was performed using IBM SPSS Statistics for Windows, Version 22.0 (IBM, Armonk, NY, USA). The correlation analyses between the variables WT% and AT% were assessed using Pearson’s and Spearman’s correlation coefficient. Normality was assessed with the Kolmogorov–Smirnov and Shapiro–Wilk tests. Friedman’s test for related samples and Tukey’s multiple comparisons test allowed detection of differences between layers for each element. To compare the behaviour of the quantitative variables considered for consistency, the Wilcoxon rank-sum test for two independent samples was used. The Chi-square test was applied to check the independence of the variables gender and smoker in relation to consistency. To study consistency as a dependent dichotomous qualitative variable, a predictive multivariate regression was applied (binary logistic regression), with the forward conditional method. In all analyses the significance level was 0.05.

## 3. Results

The results of elemental composition obtained using SEM-EDS included an EDS spectrum and a table with the quantification of each element (Figure 2). The raw results of the elemental composition of each layer of the nail plate from the samples analysed are openly available in Supplementary Materials (Figshare at https://doi.org/10.6084/m9.figshare.13399646.v1).

The correlation tests showed that the variables WT% and AT% were strongly correlated directly for every element and layer, with coefficients very close to 1. The lowest correlation value was obtained for silicon in the dorsal layer: Pearson = 0.97 and Spearman = 0.97 (*p*-values < 0.001). Therefore, the other statistical analyses were performed using only the variable WT%, because AT% provided no further information.

In total, 72 tests were conducted to assess the normality of each element in every layer by consistency. The variables did not follow a normal distribution in 72.22% of tests (*n* = 52) for Kolmogorov–Smirnov and in 80.56% (*n* = 58) for Shapiro–Wilk (*p*-values < 0.043).

Using SEM-EDS, 12 elements were identified in the samples analysed: carbon, nitrogen, oxygen, sodium, magnesium, aluminium, silicon, phosphorus, sulphur, chloride, potassium, and calcium. They were detected in the dorsal layer (Table 2), intermediate layer (Table 3), and ventral layer (Table 4), although not all the elements were detected in all the layers of the samples analysed. Carbon, oxygen and nitrogen had higher mean WT% values in the nail plates analysed (39.58 ± 1.24%, 30.41 ± 0.41% and 25.49 ± 0.86%, respectively). The other elements had much lower values, below 0.50% on average, except sulphur, at 3.63 ± 0.55% (Table 1, Table 2 and Table 3).

When the proportion of each element was compared in the dorsal, intermediate, and ventral layers, significant differences were found in the WT% values for carbon, nitrogen, phosphorus, sulphur, and calcium (*p*-values ≤ 0.01), although the relation between the layers varied depending on the element analysed. No significant differences were observed for the other elements (*p*-values > 0.104) (Table 5).

In the comparisons of all the variables considered for consistency, significant differences were detected in calcium and magnesium (*p*-values < 0.039) and indications of significance were observed in potassium (*p*-value = 0.052).

Because of this, a binary logistic regression was performed to assess the effect on consistency of gender, smoking, age, BMI, thickness, and WT% values by layers and elements. The three-step model obtained fits the observations (*p*-value = 0.538 in the Hosmer-Lemeshow test and Nagelkerke R2 = 0.355) and is useful for predicting consistency [26] (*p*-value = 0.001 in the Omnibus test, with 75.4% of cases correctly classified). Of all the predictive variables analysed, only calcium in the dorsal layer was statistically significant, with a negative coefficient (tending to be higher in hard-consistency nails than in soft-consistency nails) of −6.226 (*p*-value = 0.006). Indications of significance were detected for magnesium in the intermediate layer and potassium in the ventral layer, with positive coefficients (tending to be higher in soft-consistency nails than in hard-consistency nails) of 25.916 and 6.873, respectively (*p*-values = 0.058 and 0.089) (Table 6). The value of the model constant was 0.338. Therefore, the equation for the predictive model of nail consistency obtained by binary logistic regression was: logit(p) = 0.338 − 6.226Ca_d + 25.916Mg_i + 6.873K_v, where logit(p) = p/(1 − p), Ca_d = calcium level in the dorsal layer, Mg_i = magnesium level in the intermediate layer, K_v = potassium level in the ventral layer, and p = predictive probability for soft-consistency nails. To summarize, predictive probabilities ≤0.5 corresponded to hard-consistency nails and predictive values >0.5 corresponded to soft-consistency nails.

To test the predictive model, two additional samples of one soft-consistency and one hard-consistency nail were chosen. After applying the model obtained, nail consistency was correctly predicted in both cases.

## 4. Discussion

The four most abundant elements found were carbon, oxygen, nitrogen, and sulphur, supporting the results of earlier studies [14,15], even though the elemental analyses in other studies were performed only in the dorsal area of the nail plate rather than by layers throughout a transverse plane, as in this study. Similarly, there appeared to be no difference in the results with respect to whether the analysis was performed throughout a segment or in a specific area, whether the samples were from healthy fingernails or toenails, or the age of the participants [14,15,27].

However, examining the nails by layers allowed more in-depth analysis and established differences between them. All the most abundant elements except oxygen, and two of the least abundant elements (calcium and phosphorus) showed differences in distribution by layer. The disulphide bonds in the dorsal layer appear to be more stable than in the other layers [17]. This would keep the keratin filaments in the outermost layer, which is more exposed to external factors, more tightly packed. This circumstance, coupled with the higher level of sulphur detected in the dorsal and ventral layers, could be the result of the random distribution of keratin filaments in these layers. Random distribution would require a higher number of very stable bonds, requiring a higher number of cysteine residues to maintain cohesion inside these two layers, which are also thinner [5,21].

Calcium and magnesium are two divalent cations that are often associated in living beings because they perform important biological functions [28]. Their metabolisms are usually interrelated, and they are principal components of some tissues such as bone. This is why it was not surprising that, among all the variables analysed for nail consistency, only these two showed significant differences. Despite the differences shown by these elements, the main predictive variable in the calculation of the predictive model of nail consistency was the level of calcium in the dorsal layer, in both groups (young people and adults). Earlier studies demonstrated that the concentration of calcium in the toenails decreased significantly with age, both in men and women [28]. However, the model was not affected in the older group (adults) and the variable of level of calcium in the dorsal layer continued to be the main predictive variable.

The study of the levels of calcium showed that the dorsal layer had the largest concentration, in agreement with other studies [27]. However, studies of toughness using the scissor cutting test on each individual layer showed no differences in toughness between the dorsal and ventral layers [20]. The higher level of calcium in the dorsal layer does not appear to make this layer harder to cut. Surprisingly, the level of calcium in the dorsal layer was associated with hard-consistency nails because it was identified as the most significant predictive variable in the model, allowing hard-consistency nails to be identified as those with a higher percentage of calcium in the dorsal layer, despite the lower concentrations of this element compared to the most abundant elements (carbon, oxygen, nitrogen, and sulphur) and aluminium.

It appears logical that the considerable flattening described in the cells of the dorsal layer [18] could be associated with a higher density of desmosomes, which link cells tightly to each other, given that these bond structures were identified in all nail plate layers [29,30]. The protein isolated in the largest amount in the protein extracts from the nails is the DSP protein, a desmoplakin involved in the formation of desmosomes [12]. Another protein family present in desmosomes is that of the calcium-dependent cadherins [31]. The high levels of calcium in the dorsal layer could therefore be explained by the high presence of calcium-dependent proteins characteristic of cell–cell adhesion structures. This indicates the importance of calcium in the dorsal layer as a predictor of hard-consistency nails in the model presented.

None of the most abundant elements analysed appeared to be essential for determining nail consistency (at least with the predictive model developed), even though differences were determined in the elemental analysis of each layer. Similarly, nail plate thickness was not found to be a valid predictive variable of consistency, as described in [23], although we consider that the methodology for measuring thickness in this study is more sensitive than the micrometres normally used [16,18,19,21,32]. It is noteworthy that the values of the thickness measures in this study are lower than those obtained in previous studies [17,18]. This is probably because of the different methodological approach, given that the present study used higher image analysis sensitivity, performed measurements ex vivo (which could have reduced sources of variability in the measurements), and applied a cleaning process before image analysis (which would have removed extraneous content from under the nail plate that was not part of the nail structure). Gender was another of the variables analysed that was not useful for predicting nail consistency. However, earlier studies established differences in the amino acid content of the keratins present in the fingernails of men and women, and in the number of disulphide bonds and number of α-helical regions, β-sheets, and random coil secondary structures [33].

The predictive model presented here supports earlier studies of nail consistency determination in vivo [23,24] and is a new, useful tool for objectively determining nail consistency. Additionally, because the model was developed on healthy nail plates, it is possible to determine healthy consistency values in each individual and identify any deviations caused by various pathological states that alter the characteristics of healthy nail plates. As well as making preventive diagnosis of certain systemic and nail pathologies easier, it will open up an extensive field of practical applications. For example, in the study of medicines and doses, variations can be introduced according to the nail consistency of each individual; in nail correction processes, the most appropriate method can be used according to the consistency of the nail plate requiring treatment; and in sports, recommendations can be made to athletes according to the sport they do and their nail consistency. There are also applications for routine activities of daily life. Applying the predictive model of nail consistency coupled with high throughput SEM-EDS analysis will make it possible to automate the process and analyse a large number of samples, providing results that will make it easier to implement the practical and clinical applications.

## 5. Limitations of the Study

One of the main limitations of the study is the sample size. In particular, the number of samples available for testing the predictive model developed may seem small. However, we tested all the samples we were able to process with the available funding. Even though preparing and storing samples until analysis are not costly procedures, elemental analysis using SEM-EDS was the limiting factor in determining sample size. Moreover, increasing the number of samples to test the model, to the detriment of the number of samples available for developing the model, could have affected its reliability. Despite this, we consider that the sample size of the study made it possible to develop the model and identify its predictive variables.

A further limitation of the study was that it was not possible to measure the relative thicknesses of the three layers that make up the nail plate. Although this was not the main objective of the study, it could provide interesting data for comparison with earlier studies. This is a purely methodological limitation, because the conditions required for elemental analysis are incompatible with the conditions necessary to obtain images with the resolution needed to identify the limits between layers.

## 6. Conclusions

SEM-EDS is a useful technique to analyse elemental composition in each layer of the nail plate. The dorsal, intermediate, and ventral layers are differentiated not only in the distribution of the intermediate filaments and thickness, but also in the levels of the elements present in each layer. A predictive model of nail consistency gives health professionals better understanding of nail characteristics and, in particular, allows them to determine nail consistency from the level of calcium in the dorsal layer.

## Figures and Tables

**Table 1:** Description of the sample chosen to develop the predictive model of nail consistency. *n*, sample size; SD, standard deviation; BMI, body mass index; kg, kilograms; m^2^, square metre; µm, micrometer; Y, yes; N, no; H, hard; S, soft.

Variable	Total (SD) *n* = 57		Young Men (SD) *n* = 15		Adult Men (SD) *n* = 13		Young Women (SD) *n* = 15			Adult Women (SD) *n* = 14	
Age (years)	35.05 (±14.75)		22.40 (±3.54)		51.08 (±3.50)		20.60 (±1.68)			49.21 (±3.24)	
BMI (kg/m^2^)	25.37 (±4.18)		25.30 (±4.16)		26.53 (±3.90)		23.95 (±2.84)			25.91(±5.48)	
Thickness (µm)	558.19 (±165.40)		647.52 (±131.77)		542.33 (±128.43)		451.87 (±151.66)			591.12 (±188.37)	
Smoker	Y *n* = 20 (35.09%)	N *n* = 37 (64.91)	Y *n* = 8 (53.33)	N *n* = 7 (46.67)	Y *n* = 5 (38.46)	N *n* = 8 (61.54)	Y *n* = 3 (20.00)	N *n* = 12 (80.00)	Y *n* = 4 (28.57)		N *n* = 10 (71.43)
Consistency	H *n* = 33 (57.90%)	S *n* = 24 (42.10%)	H *n* = 8 (53.33%)	S *n* = 7 (46.67%)	H *n* = 8 (61.54%)	S *n* = 5 (38.46%)	H *n* = 8 (53.33%)	S *n* = 7 (46.67%)	H *n* = 9 (64.29%)		S *n* = 5 (35.71%)

**Table 2:** WT% values of elements in the dorsal layer. WT%, weight percentage; CONS, consistency; C, carbon; N, nitrogen; O, oxygen; Na, sodium; Mg, magnesium; Al, aluminium; Si, silicon; P, phosphorus; S, sulphur; Cl, chloride; K, potassium; Ca, calcium; H, hard; S, soft; SD, standard deviation.

		WT% Dorsal Layer											
Sample	CONS.	C	N	O	Na	Mg	Al	Si	P	S	Cl	K	Ca
33	H	40.66	25.52	29.11	0.14	0.00	0.16	0.00	0.12	3.86	0.00	0.11	0.33
44	H	41.96	25.12	27.88	0.00	0.00	0.00	0.00	0.00	4.59	0.00	0.21	0.25
45	S	41.67	24.72	28.51	0.31	0.07	0.13	0.14	0.08	3.58	0.18	0.20	0.40
52	S	39.52	24.26	30.23	0.00	0.00	1.01	0.12	0.00	4.02	0.00	0.26	0.56
61	S	42.10	25.90	26.64	0.00	0.00	0.14	0.00	0.00	4.92	0.00	0.00	0.29
62	S	39.82	27.04	29.61	0.00	0.00	0.17	0.00	0.00	3.35	0.00	0.00	0.00
63	H	42.32	22.52	24.95	0.00	0.00	0.49	0.15	0.25	7.47	0.33	0.34	1.19
90	H	39.10	27.66	30.67	0.00	0.00	0.17	0.00	0.00	2.24	0.04	0.00	0.12
98	S	39.76	27.60	29.32	0.00	0.00	0.14	0.00	0.00	3.01	0.06	0.00	0.11
103	H	39.38	28.17	29.51	0.00	0.00	0.25	0.00	0.00	2.39	0.00	0.00	0.30
106	S	38.81	27.95	30.07	0.00	0.00	0.24	0.00	0.00	2.93	0.00	0.00	0.00
122	H	38.73	26.64	30.29	0.12	0.03	0.14	0.00	0.20	3.43	0.00	0.00	0.42
124	H	40.17	26.29	30.10	0.18	0.00	0.35	0.00	0.00	2.58	0.00	0.13	0.21
135	S	38.65	26.90	32.19	0.00	0.00	0.20	0.00	0.00	2.06	0.00	0.00	0.00
139	H	40.27	25.73	27.80	0.08	0.06	0.16	0.08	0.00	5.71	0.00	0.11	0.00
158	S	36.33	28.14	32.68	0.00	0.08	0.81	0.00	0.04	1.81	0.00	0.00	0.10
192	S	38.24	27.40	31.74	0.00	0.00	0.43	0.00	0.00	2.09	0.00	0.00	0.10
196	H	41.15	27.45	27.89	0.13	0.00	0.19	0.06	0.00	2.78	0.16	0.11	0.08
210	H	38.24	27.76	31.41	0.00	0.00	0.45	0.00	0.00	2.09	0.00	0.05	0.00
282	H	45.53	22.93	27.05	0.00	0.00	0.40	0.00	0.00	3.76	0.00	0.00	0.32
351	H	40.03	26.23	29.46	0.00	0.00	0.40	0.00	0.10	3.50	0.05	0.00	0.22
368	H	40.96	25.33	29.09	0.00	0.00	0.15	0.08	0.17	3.69	0.00	0.00	0.52
413	H	36.77	21.20	37.67	1.87	0.16	0.23	0.00	0.00	1.44	0.00	0.00	0.67
501	H	39.91	26.03	30.40	0.00	0.00	0.22	0.00	0.00	3.31	0.00	0.00	0.13
576	H	39.08	26.07	31.49	0.10	0.00	0.26	0.13	0.00	2.66	0.00	0.00	0.22
577	H	41.14	17.12	28.76	0.00	0.40	5.39	0.17	0.60	4.38	0.00	1.26	0.78
586	S	41.93	24.74	26.08	0.17	0.07	0.14	0.11	0.10	6.00	0.18	0.19	0.28
77	S	40.74	25.50	30.27	0.16	0.00	0.13	0.05	0.00	2.98	0.00	0.00	0.17
78	S	33.88	30.47	33.93	0.00	0.00	0.59	0.00	0.00	1.13	0.00	0.00	0.00
86	S	45.03	23.35	24.24	0.00	0.00	0.93	0.11	0.00	6.35	0.00	0.00	0.00
87	H	37.48	29.92	29.81	0.00	0.00	0.37	0.00	0.00	2.42	0.00	0.00	0.00
95	H	41.65	24.11	27.00	0.00	0.00	0.65	0.10	0.16	5.69	0.00	0.00	0.63
100	H	42.16	24.54	28.19	0.00	0.00	0.08	0.00	0.00	4.83	0.00	0.00	0.21
102	H	35.74	30.5	31.96	0.00	0.00	0.57	0.00	0.00	1.24	0.00	0.00	0.00
112	S	39.68	24.12	33.22	0.00	0.11	1.82	0.06	0.00	0.99	0.00	0.00	0.00
114	H	37.98	27.05	32.53	0.00	0.00	0.12	0.00	0.00	2.19	0.00	0.00	0.13
156	S	42.08	23.02	30.56	0.00	0.09	0.15	0.00	0.00	3.96	0.00	0.00	0.12
170	H	42.48	22.30	29.86	0.22	0.09	0.34	0.12	0.00	4.26	0.00	0.00	0.32
184	H	45.67	20.66	28.33	0.00	0.00	0.25	0.07	0.00	4.57	0.00	0.11	0.34
280	S	38.54	25.74	33.89	0.00	0.00	0.23	0.00	0.00	1.59	0.00	0.00	0.00
363	S	41.74	24.38	30.20	0.00	0.00	0.24	0.05	0.09	3.11	0.00	0.00	0.19
76	H	42.85	22.95	30.27	0.00	0.00	0.66	0.00	0.00	3.10	0.00	0.00	0.16
83	H	42.46	23.06	31.05	0.18	0.00	0.53	0.08	0.00	2.24	0.14	0.11	0.16
238	H	42.81	23.09	30.50	0.28	0.00	0.91	0.20	0.00	2.02	0.00	0.07	0.14
259	H	44.98	21.08	29.15	0.00	0.00	0.27	0.14	0.00	3.70	0.20	0.00	0.48
266	S	41.64	23.24	30.58	0.22	0.00	0.24	0.07	0.00	3.42	0.15	0.22	0.22
278	H	46.46	18.98	30.40	0.30	0.07	0.34	0.16	0.00	2.39	0.14	0.16	0.60
297	H	41.92	22.99	30.87	0.00	0.00	0.17	0.00	0.14	3.45	0.00	0.00	0.46
326	H	42.02	23.02	30.61	0.00	0.00	0.43	0.11	0.00	3.56	0.00	0.00	0.25
340	S	43.82	22.33	30.26	0.26	0.00	0.20	0.00	0.00	2.50	0.24	0.18	0.21
342	H	42.25	23.09	30.08	0.00	0.00	0.22	0.00	0.18	3.66	0.00	0.00	0.52
441	S	43.12	22.90	29.09	0.00	0.00	0.27	0.07	0.00	4.08	0.13	0.15	0.20
458	H	42.42	23.47	28.97	0.28	0.00	0.37	0.00	0.13	3.85	0.21	0.00	0.30
599	S	38.45	23.58	32.82	0.76	0.20	1.57	0.17	0.00	2.16	0.00	0.11	0.19
600	S	38.08	25.00	32.81	0.44	0.11	0.93	0.00	0.15	1.74	0.28	0.11	0.35
601	S	39.83	25.42	30.80	0.22	0.08	0.26	0.13	0.00	3.13	0.00	0.00	0.14
121	S	54.45	16.14	24.43	0.37	0.11	0.32	0.00	0.00	3.41	0.26	0.21	0.19
Total mean ± SD		40.96 ± 3.07	24.71 ± 2.84	29.95 ± 2.34	0.12 ± 0.28	0.03 ± 0.07	0.47 ± 0.74	0.05 ± 0.06	0.04 ± 0.10	3.29 ± 1.32	0.05 ± 0.09	0.08 ± 0.18	0.25 ± 0.23

**Table 3:** WT% values of elements in the intermediate layer. WT%, weight percentage; CONS, consistency; C, carbon; N, nitrogen; O, oxygen; Na, sodium; Mg, magnesium; Al, aluminium; Si, silicon; P, phosphorus; S, sulphur; Cl, chloride; K, potassium; Ca, calcium; H, hard; S, soft; SD, standard deviation.

		WT% Intermediate Layer											
Sample	CONS.	C	N	O	Na	Mg	Al	Si	P	S	Cl	K	Ca
33	H	40.03	24.50	30.69	0.00	0.00	0.21	0.05	0.00	4.29	0.00	0.08	0.14
44	H	39.57	24.60	30.64	0.15	0.00	0.11	0.00	0.07	4.60	0.00	0.19	0.07
45	S	41.63	22.59	28.64	0.08	0.09	0.72	0.07	0.06	5.58	0.21	0.19	0.14
52	S	38.86	27.22	29.75	0.18	0.05	0.19	0.05	0.00	3.69	0.00	0.00	0.00
61	S	39.55	24.88	30.80	0.11	0.04	0.19	0.06	0.00	4.28	0.00	0.00	0.09
62	S	41.31	23.14	29.73	0.00	0.00	0.33	0.08	0.00	5.29	0.00	0.07	0.04
63	H	39.84	24.55	29.96	0.00	0.00	0.18	0.08	0.00	5.30	0.02	0.00	0.07
90	H	37.67	27.79	30.79	0.00	0.00	0.23	0.00	0.00	3.49	0.03	0.00	0.00
98	S	33.73	29.46	34.78	0.00	0.00	0.24	0.00	0.00	1.79	0.00	0.00	0.00
103	H	35.24	30.62	31.69	0.00	0.00	0.48	0.00	0.16	1.81	0.00	0.00	0.00
106	S	33.99	29.53	34.42	0.00	0.00	0.55	0.00	0.00	1.51	0.00	0.00	0.00
122	H	37.72	27.22	31.92	0.12	0.00	0.00	0.00	0.00	3.03	0.00	0.00	0.00
124	H	38.73	25.49	30.39	0.20	0.00	0.13	0.04	0.00	4.67	0.00	0.22	0.12
135	S	38.88	26.56	30.47	0.00	0.00	0.10	0.00	0.00	3.97	0.00	0.00	0.00
139	H	39.79	25.80	29.08	0.00	0.00	0.17	0.07	0.00	5.09	0.00	0.00	0.00
158	S	36.53	27.12	33.03	0.00	0.00	0.12	0.00	0.00	3.10	0.00	0.00	0.10
192	S	39.56	24.97	31.01	0.19	0.08	0.15	0.09	0.00	3.64	0.00	0.11	0.19
196	H	35.31	29.87	32.53	0.00	0.00	0.52	0.00	0.00	1.78	0.00	0.00	0.00
210	H	39.39	26.16	29.34	0.00	0.00	0.14	0.05	0.00	4.84	0.08	0.00	0.00
282	H	38.34	27.14	30.88	0.00	0.00	0.22	0.00	0.00	3.42	0.00	0.00	0.00
351	H	37.83	27.47	31.19	0.00	0.00	0.38	0.00	0.00	3.13	0.00	0.00	0.00
368	H	38.13	27.15	30.94	0.00	0.00	0.12	0.00	0.00	3.65	0.00	0.00	0.00
413	H	36.10	23.83	35.92	0.86	0.09	0.16	0.06	0.00	2.74	0.00	0.00	0.24
501	H	39.71	26.08	28.65	0.00	0.00	0.16	0.06	0.00	5.33	0.00	0.00	0.00
576	H	43.24	23.33	25.41	0.00	0.00	0.64	0.10	0.00	7.27	0.00	0.00	0.00
577	H	39.25	26.16	29.98	0.00	0.07	0.12	0.00	0.15	4.28	0.00	0.00	0.00
586	S	40.12	25.9	28.27	0.13	0.02	0.10	0.05	0.00	5.18	0.12	0.09	0.02
77	S	39.53	26.32	29.43	0.15	0.00	0.11	0.06	0.00	4.39	0.00	0.00	0.00
78	S	39.55	26.24	29.40	0.00	0.00	0.19	0.00	0.00	4.50	0.00	0.00	0.11
86	S	35.11	28.61	33.77	0.00	0.00	0.80	0.03	0.00	1.69	0.00	0.00	0.00
87	H	34.08	29.45	34.62	0.00	0.00	0.44	0.00	0.00	1.41	0.00	0.00	0.00
95	H	36.95	27.01	32.87	0.00	0.00	0.68	0.00	0.00	2.48	0.00	0.00	0.00
100	H	41.00	24.79	26.38	0.00	0.00	0.50	0.09	0.00	6.72	0.00	0.23	0.30
102	H	37.68	26.39	32.37	0.00	0.00	0.37	0.00	0.00	3.18	0.00	0.00	0.00
112	S	41.13	22.58	32.93	0.00	0.17	1.70	0.10	0.00	1.40	0.00	0.00	0.00
114	H	36.19	27.86	33.32	0.00	0.00	0.16	0.00	0.00	2.46	0.00	0.00	0.00
156	S	41.8	23.41	28.98	0.00	0.09	0.21	0.00	0.00	5.35	0.00	0.00	0.15
170	H	38.79	25.22	30.84	0.00	0.00	0.19	0.06	0.00	4.76	0.00	0.00	0.15
184	H	43.15	21.51	27.96	0.00	0.00	0.29	0.06	0.10	6.47	0.00	0.14	0.32
280	S	39.31	25.09	31.10	0.00	0.00	0.24	0.00	0.00	4.16	0.00	0.00	0.09
363	S	39.98	23.39	30.96	0.12	0.00	0.24	0.08	0.00	4.94	0.00	0.12	0.17
76	H	41.81	22.34	28.76	0.00	0.00	0.3	0.00	0.00	6.79	0.00	0.00	0.00
83	H	40.19	23.85	29.62	0.21	0.00	0.18	0.03	0.00	5.39	0.26	0.20	0.08
238	H	41.63	23.63	28.71	0.00	0.00	0.33	0.09	0.00	5.43	0.00	0.00	0.18
259	H	40.06	24.22	30.13	0.00	0.00	0.22	0.07	0.00	5.13	0.00	0.00	0.16
266	S	38.90	24.53	31.41	0.17	0.00	0.17	0.00	0.00	4.34	0.00	0.25	0.24
278	H	41.03	22.95	29.27	0.17	0.00	0.33	0.07	0.00	6.10	0.00	0.00	0.08
297	H	38.83	24.43	32.18	0.18	0.00	0.19	0.00	0.00	4.19	0.00	0.00	0.00
326	H	39.36	24.57	30.54	0.13	0.03	0.22	0.05	0.00	5.07	0.00	0.00	0.03
340	S	40.53	23.42	29.21	0.18	0.00	0.28	0.06	0.11	5.70	0.00	0.20	0.31
342	H	38.49	25.25	31.39	0.00	0.00	0.22	0.07	0.00	4.46	0.00	0.00	0.13
441	S	38.68	25.17	31.12	0.00	0.00	0.20	0.00	0.00	4.62	0.00	0.00	0.21
458	H	40.81	23.43	28.87	0.25	0.00	0.56	0.00	0.00	5.92	0.15	0.00	0.00
599	S	42.02	23.51	27.24	0.10	0.00	0.35	0.09	0.05	6.17	0.12	0.11	0.25
600	S	36.71	25.83	32.16	0.42	0.00	0.65	0.00	0.00	3.54	0.43	0.13	0.13
601	S	38.37	25.48	30.84	0.17	0.00	0.24	0.10	0.00	4.50	0.17	0.12	0.00
121	S	54.24	15.82	23.15	0.38	0.06	0.22	0.11	0.00	5.41	0.31	0.22	0.09
Total mean ± SD		39.23 ± 2.98	25.36 ± 2.43	30.53 ± 2.26	0.08 ± 0.15	0.01 ± 0.03	0.31 ± 0.26	0.04 ± 0.04	0.01 ± 0.04	4.27 ± 1.46	0.03 ± 0.09	0.05 ± 0.08	0.08 ± 0.09

**Table 4:** WT% values of elements in the ventral layer. WT%, weight percentage; CONS, consistency; C, carbon; N, nitrogen; O, oxygen; Na, sodium; Mg, magnesium; Al, aluminium; Si, silicon; P, phosphorus; S, sulphur; Cl, chloride; K, potassium; Ca, calcium; H, hard; S, soft; SD, standard deviation.

		WT% Ventral Layer											
Sample	CONS.	C	N	O	Na	Mg	Al	Si	P	S	Cl	K	Ca
33	H	37.77	26.08	31.87	0.00	0.00	0.22	0.05	0.00	3.79	0.00	0.10	0.13
44	H	36.44	26.66	33.15	0.00	0.00	0.08	0.00	0.00	3.51	0.00	0.15	0.00
45	S	40.02	26.58	28.58	0.09	0.00	0.26	0.11	0.08	3.94	0.14	0.07	0.15
52	S	36.38	30.72	30.04	0.15	0.00	0.27	0.00	0.00	2.44	0.00	0.00	0.00
61	S	38.07	27.74	30.74	0.00	0.00	0.11	0.00	0.00	3.34	0.00	0.00	0.00
62	S	39.95	26.72	31.48	0.00	0.00	0.33	0.00	0.00	1.51	0.00	0.00	0.00
63	H	41.90	23.91	29.04	0.00	0.00	0.20	0.07	0.00	4.88	0.00	0.00	0.00
90	H	32.78	31.40	34.16	0.00	0.00	0.07	0.00	0.00	1.60	0.00	0.00	0.00
98	S	36.34	29.03	31.54	0.00	0.00	0.29	0.00	0.00	2.81	0.00	0.00	0.00
103	H	32.57	33.54	31.83	0.00	0.00	0.18	0.00	0.00	1.88	0.00	0.00	0.00
106	S	35.85	28.78	32.30	0.00	0.00	0.07	0.05	0.00	2.89	0.00	0.00	0.07
122	H	36.57	28.97	31.62	0.15	0.00	0.26	0.00	0.00	2.42	0.00	0.00	0.00
124	H	35.08	29.29	33.03	0.17	0.00	0.49	0.00	0.00	1.93	0.00	0.01	0.00
135	S	34.49	30.39	33.06	0.00	0.00	0.15	0.00	0.00	1.91	0.00	0.00	0.00
139	H	35.62	32.14	29.82	0.00	0.00	0.77	0.00	0.00	1.65	0.00	0.00	0.00
158	S	40.68	24.87	28.56	0.00	0.00	0.77	0.07	0.00	5.04	0.00	0.00	0.00
192	S	35.78	28.35	33.26	0.14	0.00	0.20	0.03	0.00	2.16	0.00	0.02	0.06
196	H	31.93	35.23	30.49	0.00	0.08	1.56	0.00	0.00	0.71	0.00	0.00	0.00
210	H	36.27	29.33	31.44	0.00	0.00	0.09	0.00	0.00	2.87	0.00	0.00	0.00
282	H	33.22	33.39	31.70	0.00	0.00	0.68	0.00	0.00	1.01	0.00	0.00	0.00
351	H	37.08	30.24	30.71	0.00	0.00	0.12	0.00	0.00	1.84	0.00	0.00	0.00
368	H	36.93	27.31	31.60	0.27	0.11	0.48	0.00	0.00	2.79	0.25	0.10	0.15
413	H	37.46	17.07	40.98	1.30	0.18	0.12	0.07	0.25	1.68	0.00	0.27	0.62
501	H	37.99	27.97	30.76	0.00	0.00	0.18	0.05	0.00	3.04	0.00	0.00	0.00
576	H	51.51	9.07	32.01	0.00	0.00	4.86	0.00	0.00	2.55	0.00	0.00	0.00
577	H	46.63	21.78	23.41	0.00	0.14	1.39	0.12	0.13	6.40	0.00	0.00	0.00
586	S	36.49	29.17	31.91	0.12	0.00	0.13	0.00	0.00	2.18	0.00	0.00	0.00
77	S	38.32	26.88	31.40	0.00	0.00	0.17	0.00	0.00	3.24	0.00	0.00	0.00
78	S	36.19	30.66	30.03	0.00	0.00	1.50	0.00	0.00	1.61	0.00	0.00	0.00
86	S	34.44	30.10	33.13	0.00	0.00	0.47	0.04	0.00	1.81	0.00	0.00	0.00
87	H	35.25	30.36	32.00	0.00	0.00	0.35	0.00	0.00	2.04	0.00	0.00	0.00
95	H	35.52	28.46	33.78	0.00	0.00	0.31	0.00	0.00	1.92	0.00	0.00	0.00
100	H	42.34	21.37	22.78	0.00	0.17	7.83	0.12	0.00	5.39	0.00	0.00	0.00
102	H	32.67	33.28	31.87	0.00	0.00	1.37	0.00	0.00	0.81	0.00	0.00	0.00
112	S	40.46	22.89	33.90	0.00	0.13	1.55	0.09	0.00	0.98	0.00	0.00	0.00
114	H	32.81	30.66	35.01	0.00	0.00	0.39	0.00	0.00	1.13	0.00	0.00	0.00
156	S	39.58	25.77	30.02	0.00	0.00	0.20	0.00	0.00	4.31	0.00	0.00	0.13
170	H	42.42	23.28	28.43	0.00	0.00	0.30	0.08	0.00	5.48	0.00	0.00	0.00
184	H	42.35	22.02	28.14	0.00	0.00	0.28	0.10	0.00	6.92	0.00	0.00	0.19
280	S	36.97	26.11	33.32	0.00	0.00	0.33	0.00	0.00	3.28	0.00	0.00	0.00
363	S	42.73	22.72	29.52	0.09	0.00	0.18	0.07	0.00	4.43	0.00	0.15	0.11
76	H	43.24	23.73	27.76	0.00	0.00	0.30	0.00	0.00	4.85	0.00	0.00	0.12
83	H	39.50	25.17	29.62	0.17	0.00	0.28	0.07	0.00	4.96	0.17	0.06	0.00
238	H	35.97	28.32	32.06	0.17	0.00	0.20	0.00	0.00	3.28	0.00	0.00	0.00
259	H	40.19	24.23	29.79	0.27	0.00	0.23	0.08	0.00	5.05	0.06	0.03	0.08
266	S	36.10	26.12	33.68	0.23	0.00	0.12	0.00	0.00	3.41	0.14	0.19	0.00
278	H	38.27	25.23	31.05	0.26	0.00	0.30	0.10	0.00	4.79	0.00	0.00	0.00
297	H	39.14	24.72	31.48	0.00	0.00	0.26	0.00	0.00	4.40	0.00	0.00	0.00
326	H	42.52	23.84	29.85	0.00	0.00	0.36	0.00	0.00	3.23	0.00	0.00	0.20
340	S	41.07	23.85	28.68	0.11	0.00	0.30	0.00	0.00	6.00	0.00	0.00	0.00
342	H	38.93	25.50	31.15	0.00	0.00	0.21	0.06	0.00	4.15	0.00	0.00	0.00
441	S	40.47	25.14	28.27	0.00	0.00	0.30	0.08	0.00	5.60	0.00	0.00	0.14
458	H	42.52	23.04	26.94	0.21	0.13	1.39	0.09	0.00	5.32	0.37	0.00	0.00
599	S	39.83	25.20	29.14	0.00	0.00	0.28	0.00	0.00	5.34	0.00	0.00	0.21
600	S	43.65	18.03	27.00	0.28	0.34	3.70	0.14	0.00	4.78	0.74	0.59	0.75
601	S	40.03	24.97	29.65	0.18	0.07	0.27	0.08	0.00	4.45	0.12	0.16	0.00
121	S	52.71	17.73	23.62	0.43	0.05	0.21	0.09	0.00	4.40	0.40	0.26	0.09
Total mean ± SD		38.56 ± 4.16	26.41 ± 4.53	30.74 ± 2.89	0.08 ± 0.19	0.02 ± 0.06	0.67 ± 1.27	0.08 ± 0.04	0.01 ± 0.04	3.34 ± 1.58	0.04 ± 0.13	0.04 ± 0.10	0.06 ± 0.14

**Table 5:** Comparison of WT% values of elements by layer. *, significant difference.

		Dorsal–Intermediate	Dorsal–Ventral	Intermediate–Ventral
Element	Friedman (*p*-Value)	Tukey (*p*-Value)		
Carbon	<0.001 *	<0.001 *	<0.001 *	0.909
Nitrogen	<0.001 *	0.275	<0.001 *	<0.001 *
Phosphorus	0.01 *	0.174	0.161	0.606
Sulphur	<0.001 *	<0.001 *	0.708	<0.001 *
Calcium	<0.001 *	<0.001 *	<0.001 *	0.013

**Table 6:** WT% values for the three predictive variables of the model. WT%, weight percentage; SD, standard deviation; H, hard; S, soft.

Layer	Consistency	WT% Calcium (SD)	WT% Magnesium (SD)	WT% Potassium (SD)
Dorsal	H	0.32 (±0.26)	0.02 (±0.08)	0.08 (±0.23)
	S	0.16 (±0.15)	0.04 (±0.06)	0.07 (±0.09)
Intermediate	H	0.07 (±0.09)	0.01 (±0.02)	0.04 (±0.07)
	S	0.10 (±0.09)	0.03 (±0.04)	0.07 (±0.08)
Ventral	H	0.05 (±0.12)	0.02 (±0.05)	0.02 (±0.06)
	S	0.07 (±0.16)	0.02 (±0.07)	0.06 (±0.13)

## Data Availability

The data presented in this study are available in Supplementary Materials at https://doi.org/10.6084/m9.figshare.13399646.v1.

## Supplementary Materials

The SEM-EDS data presented in this study are openly available in Figshare at https://doi.org/10.6084/m9.figshare.13399646.v1.

## References

[1] De Berker D. (2013). Nail anatomy. Clin. Dermatol..

[2] Fleckman P., Allan C. (2001). Surgical anatomy of the nail unit. Dermatol. Surg..

[3] Haneke E. (2006). Surgical anatomy of the nail apparatus. Dermatol. Clin..

[4] Fleckman P., Jaeger K., Silva K.A., Sundberg J.P. (2013). Comparative anatomy of mouse and human nail units. Anat. Rec. Hoboken.

[5] Runne U., Orfanos C.E. (1981). The human nail. Structure, growth and pathological changes. Curr. Probl. Derm..

[6] Gniadecka M., Nielsen O.F., Christensen D.H., Wulf H.C. (1998). Structure of water, proteins, and lipids in intact human skin, hair, and nail. J. Investig. Dermatol..

[7] Schweizer J., Bowden P.E., Coulombe P.A., Langbein L., Lane E.B., Magin T.M., Maltais L., Omary M.B., Parry D.A.D., Rogers M.A. (2006). New consensus nomenclature for mammalian keratins. J. Cell Biol..

[8] Steinert P.M., Idler W.W., Zimmerman S.B. (1976). Self-assembly of bovine epidermal keratin filaments in vitro. J. Mol. Biol..

[9] Perrin C. (2007). Expression of follicular sheath keratins in the normal nail with special reference to the morphological analysis of the distal nail unit. Am. J. Dermatopathol..

[10] Perrin C., Langbein L., Schweizer J. (2004). Expression of hair keratins in the adult nail unit: An immunohistochemical analysis of the onychogenesis in the proximal nail fold, matrix and nail bed. Br. J. Dermatol..

[11] Lynch M.H., O’Guin W.M., Hardy C., Mak L., Sun T.T. (1986). Acidic and basic hair/nail (“hard”) keratins: Their colocalization in upper cortical and cuticle cells of the human hair follicle and their relationship to “soft” keratins. J. Cell Biol..

[12] Rice R.H., Xia Y., Alvarado R.J., Phinney B.S. (2010). Proteomic analysis of human nail plate. J. Proteome Res..

[13] Strnad P., Usachov V., Debes C., Gräter F., Parry D.A.D., Bishr Omary M. (2011). Unique amino acid signatures that are evolutionarily conserved distinguish simple-type, epidermal and hair keratins. J. Cell Sci..

[14] Laubé F., Poupon A., Zinck P., Goymann C.M., Reichl S., Rataj V.N. (2019). Physicochemical investigations of native nails and synthetic models for a better understanding of surface adhesion of nail lacquers. Eur. J. Pharm. Sci..

[15] Pinteala T., Chiriac A.E., Rosca I., Larese Filon F., Pinteala M., Chiriac A., Podoleanu C., Stolnicu S., Coros M.F., Coroaba A. (2016). Nail damage (severe onychodystrophy) induced by acrylate glue: Scanning electron microscopy and energy dispersive x-ray investigations. Skin Appendage Disord..

[16] Young R.W., Newman S.B., Capott R.J. (1965). Strength of Fingernails. J. Invest. Dermatol..

[17] Baraldi A., Jones S.A., Guesne S., Traynor M.J., McAuley W.J., Brown M.B., Murdan S. (2015). Human nail plate modifications induced by onychomycosis: Implications for topical therapy. Pharm. Res..

[18] De Berker D., Mawhinney B., Sviland L. (1996). Quantification of regional matrix nail production. Br. J. Dermatol..

[19] Johnson M., Shuster S. (1994). Determinants of nail thickness and length. Br. J. Dermatol..

[20] Farren L., Shayler S., Ennos A.R. (2004). The fracture properties and mechanical design of human fingernails. J. Exp. Biol..

[21] Kobayachi Y., Miyamoto M., Sugibayashi K., Morimoto Y. (1999). Drug Permeation through the Three Layers of the Human Nail Plate. J. Pharm. Pharmacol..

[22] Shemer A., Daniel C.R.I. (2013). Common nail disorders. Clin. Dermatol..

[23] Pico A.M.P., Verjano E., Mayordomo R. (2017). Relation between nail consistency and incidence of ingrown toenails in young male runners. J. Am. Podiatr. Med. Assoc..

[24] Pico A.M.P., Álvarez E.M., Cervigón N.C., Acevedo R.M. (2019). Importance of Preexisting Physical Factors in the Development of Dermatological and Muscular Lesions During Hiking. Int. J. Low. Extrem. Wounds.

[25] Takaku Y., Takehara S., Suzuki C., Suzuki H., Shimomura M., Hariyama T. (2020). In situ elemental analyses of living biological specimens using ‘NanoSuit’ and EDS methods in FE-SEM. Sci. Rep..

[26] Canela M.A. (2007). Cómo hacer una Regresión Logística con SPSS “paso a paso” (I). Fabis.

[27] Robson J.R.K., Brooks G.J. (1974). The distribution of calcium in fingernails from healthy and malnourished children. Clin. Chim. Acta.

[28] Ohgitani S., Fujita T., Fujii Y., Hayashi C., Nishio H. (2005). Nail calcium and magnesium content in relation to age and bone mineral density. J. Bone Miner. Metab..

[29] Caputo R., Gasparini G., Contini D. (1982). A freeze-fracture study of the human nail plate. Arch. Dermatol. Res..

[30] Parent D., Achten G., Vanhoof F.S. (1985). Ultrastructure of the normal human nail. Am J Dermatopathol.

[31] Frank J., Friedman P.B.C., Ahmad W., Panteleyev A.A., Aita V.M., Christiano A.M. (2001). Characterization of the desmosomal cadherin gene family: Genomic organization of two desmoglein genes on human chromosome 18q12. Exp. Dermatol..

[32] Langford D.T., Burke C., Robertson K. (1989). Risk factors in onychocryptosis. Br. J. Surg..

[33] Brzózka P., Kolodziejski W. (2017). Sex-related chemical differences in keratin from fingernail plates: A solid-state carbon-13 NMR study. RSC Adv..

